# Synthesis and hygrothermal properties of capric–myristic acid/diatomite-based composite phase change materials for thermal energy storage

**DOI:** 10.1039/d5ra06392b

**Published:** 2025-10-16

**Authors:** Meng Zhou, Qi Feng, Xiuli Sun, Yukuan Wang, Jing Deng, Hongyi Liang

**Affiliations:** a School of Green Mining and Resource Engineering, Liaoning Petrochemical University Fushun 113001 China 1172710966@qq.com; b School of Environmental and Safety Engineering, Liaoning Petrochemical University Fushun 113001 China

## Abstract

In this paper, diatomite (DE) served as the supporting material, and capric acid–myristic acid (CA–MA) was utilized as the phase change material (PCM) to synthesize CA–MA/DE composite PCM *via* the vacuum impregnation method. Leak experimental results indicated that the optimal weight ratio of CA–MA was identified as 40%. The chemical compatibility, thermal properties, and stability of CA–MA/DE were evaluated. The phase temperature and latent heat of CA–MA/DE were obtained as 22.21 °C and 74.07 J g^−1^, respectively. To study the feasibility of CA–MA/DE in energy-efficient buildings, CA–MA/DE was used to replace sand of equivalent quality to prepare the CA–MA/DE-based gypsum mortar (CA–MA/DE&GM). The results showed that the replacement rate of CA–MA/DE was determined as 30%, and the thermal properties and humidity regulation performance of CA–MA/DE&GM were significantly enhanced due to the addition of the CA–MA/DE. Notably, the digital image correlation (DIC) technique was employed to investigate the crack evolution mechanism of CA–MA/DE&GM30 (CA–MA/DE content was 30%) over 100 thermal cycles. The novel CA–MA/DE&GM30 can adjust the environmental temperature and humidity simultaneously, which has considerable potential for energy conservation in buildings.

## Abstract 2

This study use diatomite (DE) and capric–myristic acid (CA–MA) to prepare the novel CA–MA/DE-based phase change mortar with temperature and humidity control functions to improve energy utilization and environmental comfort.

## Graphical abstract

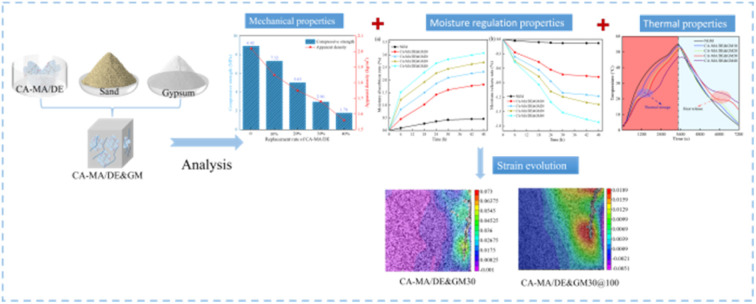

## 1 Introduction

The comfort of the living environment is closely linked to both the temperature and relative humidity (RH).^1^ For a long time, using mechanical equipment such as humidifiers and air conditioners to regulate indoor temperature and RH has caused a large amount of energy consumption and brought air quality issues broadly affecting the health of residents.^2–4^ In addition, the extensive utilization of fossil fuels leads to substantial greenhouse gas emissions, which is not conducive to environmental protection.^5^ Energy consumption in buildings accounts for approximately 40% of total energy consumption, and it has become more significant to realize low-carbon development for reducing wasted energy.^6,7^ It is crucial to develop a method that effectively enhances the comfort of the built environment and achieves energy reduction targets. The moisture absorption and heat storage can be automatically realized by using multifunctional materials in temperature and humidity regulation method. Humidity control materials (HCMs) and PCMs are widely applied in the energy-efficient building field.^8,9^ However, the single utilization of HCM or PCM is difficult to improve environmental temperature and humidity simultaneously.^10,11^ The combination of HCMs and PCMs to develop novel composite energy-saving materials with the temperature and humidity adjustment functions has become a new hot point.

PCMs can store and release heat during the phase change process with near-constant temperature, thereby effectively enhancing energy efficiency, which is extensively utilized in energy-efficient buildings, waste heat recovery systems, and solar energy applications.^12–14^ There are many kinds of PCMs, which can be categorized into organic and inorganic PCMs based on the chemical composition. Organic PCMs (paraffin,^15^ lauric acid,^16^ and lauryl alcohol,^17^*etc.*) offer superior energy storage density, enhanced stability, reduced corrosion susceptibility, and a lower cost compared to inorganic PCMs. Meanwhile, organic PCMs and building materials have better compatibility and can be used in energy-saving buildings together, which has been proven in masonry bricks,^18^ concrete,^19,20^ gypsum board,^21^ and cement mortar.^22^ Most single PCM has lower thermal conductivity, a relatively constant temperature range, and limited energy storage density. Eutectic PCMs can achieve better phase change latent heat and energy storage density by optimizing the component distribution ratio, and expand its application range, such as capric–stearic acid,^23^ capric acid–ethyl alcohol,^24^ and capric–lauric acid.^25^ However, the efficient utilization of eutectic PCMs was affected by the leakage problem, which should be encapsulated effectively before use. The common encapsulation methods include porous material adsorption, microencapsulation, and macroencapsulation.^26–28^ Significantly, PCMs can be forced into the pores of the mineral materials through the porous adsorption method in a vacuum environment, thus inhibiting the leakage issues, such as DE,^29^ expanded perlite,^30^ bentonite,^4^ and expanded vermiculite.^31^

In addition to temperature control, the moisture regulation performance of energy-saving building materials also plays an important role in controlling environmental humidity fluctuations and optimizing energy utilization rates. HCMs can automatically sense the moisture changes in the environment without consuming energy, effectively managing the absorption and release of moisture content, which have become important measures for improving the humidity adjustment ability of building materials.^32^ Among them, porous mineral materials (zeolite,^33^ kaolin,^34^ and sepiolite,^35^*etc.*) can reduce the fluctuation of RH due to the richer pore size characteristics, larger pore volume, and excellent adsorption ability, which are widely applied in energy-efficient buildings. DE is a natural moisture regulation material that has been widely considered in the field of building humidity adjustment due to its higher porosity, extensive specific surface area, stronger adsorption capacity, better chemical inertness, and lower price. In recent years, research on integrating mineral-based HCMs or PCMs into building materials to develop energy-saving materials has made great progress. Wadee *et al.* were impregnating paraffin into perlite to prepare composite PCMs to replace fine aggregate in cement mortar. It was found that the compressive strength of mortar decreased by 39.7–48.5% when the PCMs content was 50%.^36^ Wang *et al.* used capric–stearic acid/expanded perlite composite PCMs as a substitute for sand in mixed aggregate to prepare thermal mortar.^37^ The results found that the specific heat capacity of thermal mortar gradually enhances as the composite PCMs content increases, while the thermal conductivity gradually declines. Cunha *et al.* integrated PCM into mortar to develop a functional gypsum-based mortar and examined its water absorption.^38^ The results showed that the capillary and soaking water absorption of the mortar decreased by about 65% and 30%, respectively, with the addition of 20% PCM, without affecting its usability. It is important to note that the majority of researchers concentrate on the mechanical, humidity control, and thermal properties of mineral-based energy-saving mortars, whereas significantly less attention has been directed toward the strain evolution mechanism of mortars by the influence of composite PCMs.

In this study, the CA–MA/DE composite PCM was prepared through the vacuum impregnation technique. The chemical compatibility, thermal performance, and stability of the CA–MA/DE were characterized. The results demonstrated that prepared CA–MA/DE exhibits higher latent heat, suitable phase change temperature, and larger loading capacity. Besides, the humidity regulation ability, heat storage and release capacity, and mechanical capabilities of CA–MA/DE&GM were systematically studied. Meanwhile, the DIC technique was utilized to investigate the strain evolution of CA–MA/DE&GM30 during loading, aiming to elucidate the anti-damage mechanism under practical applications. The results show that axial strain has a significant effect on the strength of CA–MA/DE–GM30, and this discovery provides a theoretical basis for the study, evaluation, and reinforcement of the deformation properties of gypsum mortar. The CA–MA/DE&GM30 has obvious potential for application in energy conservation buildings, which demonstrates better thermal storage and moisture absorption ability.

## 2 Experiments

### 2.1 Materials

DE (200 mesh) was obtained from Linjiang Yuantong New Material Company, and the main chemical composition was about 89% amorphous SiO_2_. CA (C_10_H_20_O_2_, AR, Sinopharm Chemical Reagent Co., Ltd.) and MA (C_14_H_28_O_2_, AR, Sinopharm Chemical Reagent Co., Ltd.) were used as PCMs. The sand was purchased from Xiamen Ace Standard Sand Co., Ltd. The gypsum powder was supplied by Shandong Longbang Gypsum Products Co., Ltd., and was employed as the cementitious material for preparing GM. The tap water was taken from the laboratory, and all chemical reagents were analytical reagent grade and could be applied directly.

### 2.2 Experimental procedure

#### 2.2.1 Preparation of CA–MA binary eutectic mixture

The phase change temperature and latent heat of CA and MA were obtained *via* differential scanning calorimetry (DSC) tests, and the mass ratios of the components within the CA–MA eutectic mixture were predicted utilizing the second law of thermodynamics and Schröder equation.^39^ According to the calculation formula, the mass fraction of CA and MA was determined to be 75 : 25. Based on the mass ratio, the CA–MA binary mixture was weighed and mixed in a beaker, and prepared by the melting–blending method at the constant water bath temperature of 80 °C, and stirred for 30 min. Finally, the sample was placed at room temperature for cooling, and the obtained CA–MA was sealed for later use.

#### 2.2.2 Synthesis of the CA–MA/DE composite

To ensure the stability of the CA–MA/DE composite, it is necessary to determine the maximum loading amount of CA–MA. A series of CA–MA/DE composites was designed *via* the vacuum impregnation method. First, 4 g CA–MA and 6 g DE were weighed and mixed into a conical flask, and then heated at 80 °C thermostat water bath for 10 min. Then, the conical flask was connected by a suction pump to prepare the CA–MA/DE (40% CA–MA content) at approximately −0.1 MPa for 30 min. The prepared sample was placed on the filter paper at 50 °C to remove the excess CA–MA on the surface of DE. Meanwhile, the other CA–MA/DE composites with different proportions were prepared using the same method and named 20%, 30%, 50%, and 60%, respectively.

**Fig. 1:**
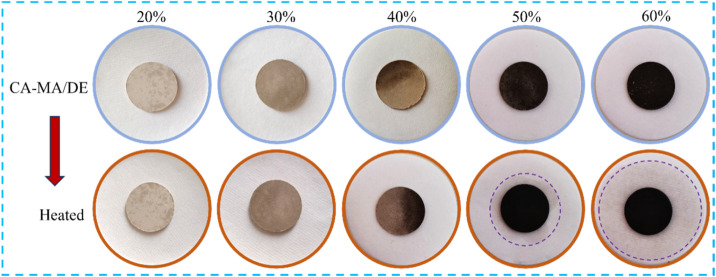
The maximum adsorption capacity of CA–MA/DE.

The leakage experiment was used to determine the maximum absorption content of CA–MA into DE and assess the form stability of the CA–MA/DE composites. All samples were pressed into sheets and then heated to 50 °C for 1 h, and the exudation stability of the CA–MA/DE composites was shown in Fig. 1. When the CA–MA ratio was between 20% and 40%, no leakage phenomenon was observed, and the CA–MA/DE composites maintained good morphological stability. When the CA–MA ratio reached 50%, the obvious leakage phenomenon was observed. It is worth noting that the leakage area expanded with the increase of the CA–MA mass fraction, and a larger seepage circle appeared at 60%. The results indicated that the optimal load ratio of CA–MA was determined to be 40%, and the CA–MA/DE was prepared according to the ratio. Meanwhile, the CA–MA/DE was heated from 10 °C to 60 °C and then cooled to 10 °C to determine the reliability for long-term applications, and the process was repeated 100 times in total. The sample after 100 phase change cycles was named CA–MA/DE-1.

#### 2.2.3 Manufacture of the CA–MA/DE&GM

**Fig. 2:**
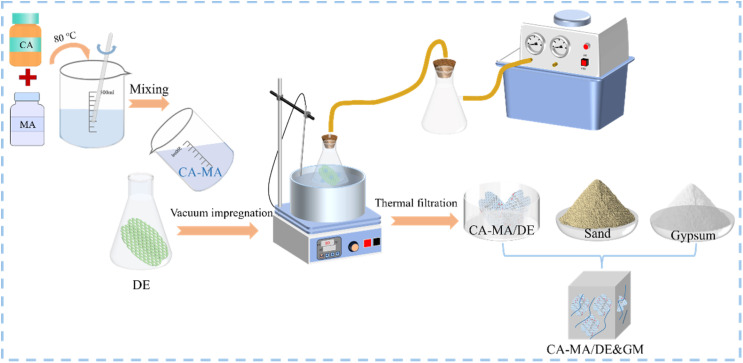
The preparation process of the CA–MA/DE&GM.

A series of GMs with different CA–MA/DE proportions were fabricated by replacing sand with equal mass (0, 10%, 20%, 30%, and 40%). Fig. 2 shows the schematic illustration of the synthesis process for the NGM and CA–MA/DE&GM, where “NGM” represents normal GM, while “CA–MA/DE&GM” stands for the CA–MA/DE-based GM. First, each component was weighed and mixed in the blender according to the proportion, and the mixtures were stirred at low speed for 2 min to mix thoroughly. Then, the water was added, and the mixtures were first stirred at the same low speed for 1 min and then at high speed for 2 min until the uniform GM mixture was obtained. Finally, we poured the slurry into the mold and put it in a constant-temperature incubator. The NGM and CA–MA/DE&GM with different CA–MA/DE contents were cured at 20 °C for 24 h, and the samples were continued to be kept for 7 days after demolding. The mixed proportion of NGM and CA–MA/DE&GM with different CA–MA content (the numbers represent different replacement ratios) is shown in Table 1.

**Table 1:** The mix design of CA–MA/DE&GM

Sample	Gypsum (kg m^−3^)	Sand (kg m^−3^)	CA–MA/DE (kg m^−3^)	Water (kg m^−3^)
NGM	500	1520	0	300
CA–MA/DE&GM10	500	1368	152	330
CA–MA/DE&GM20	500	1216	304	365
CA–MA/DE&GM30	500	1064	456	400
CA–MA/DE&GM40	500	912	608	435

### 2.3 Characterization

The chemical compatibility of CA–MA/DE was investigated using Fourier transform infrared spectroscopy (FTIR, Bruker TENSOR27) at a resolution of 4 cm^−1^ with the range of 4000–400 cm^−1^. The thermal storage capacity and cycling reliability of samples were assessed through differential scanning calorimetry (DSC, Q20) under a nitrogen atmosphere, in which the temperature scanning range was from 0 to 100 °C and the rate was 5 °C min^−1^. One thermal cycling process includes heating the CA–MA/DE from 10 to 60 °C and then cooling it to 10 °C, which is repeated 100 times in total. The thermogravimetric analysis (TGA, Q600) was used to measure the thermal stability of DE, CA–MA, and CA–MA/DE under a nitrogen atmosphere at a 10 °C min^−1^ heating rate from room temperature to 400 °C. The surface temperature variations of CA–MA and CA–MA/DE were recorded by the infrared thermal imager A700. CA–MA and CA–MA/DE were compressed into circular sheets of the same size, which were put into a mold made of foil to ensure even heating and avoid leakage during the experiment process. The samples were placed on a graphene constant temperature heating plate during the heating and cooling process. The moisture control experiments of the samples were conducted using a desiccator in constant temperature and humidity conditions. It is 100% RH for high humidity environments and 11% RH for low humidity environments. The thermal energy storage/release properties of NGM and CA–MA/DE&GM with different CA–MA/DE contents were characterized using a self-made device, which includes five PT100 thermocouples and a paperless recorder (KSA06BOR). The compressive strength of NGM and CA–MA/DE&GM with different CA–MA/DE contents was studied using the microcomputer-controlled electro-hydraulic servo universal testing machine (TAW-2000) with a loading rate of 0.05 MPa s^−1^. The apparent density was calculated based on the volume and dried mass of samples with dimensions of 70.7 × 70.7 × 70.7 mm^3^. The morphology and microstructure of DE, CA–MA/DE, NGM, and CA–MA/DE&GM30 were observed by scanning electron microscopy (SEM, FEI Quanta-200), and the samples were coated with gold before testing. The crack propagation and principal strain evolution mechanism of NGM and CA–MA/DE&GM30 in compressive strength experiments were investigated by the DIC technique. It consists of two CCD cameras with a resolution of 2448 × 2048 pixels, an A/D converter, and a PC terminal. The high-speed camera records at 10 Hz, capturing images at 2 frames per s.

## 3 Results and discussion

### 3.1 Chemical compatibility

**Fig. 3:**
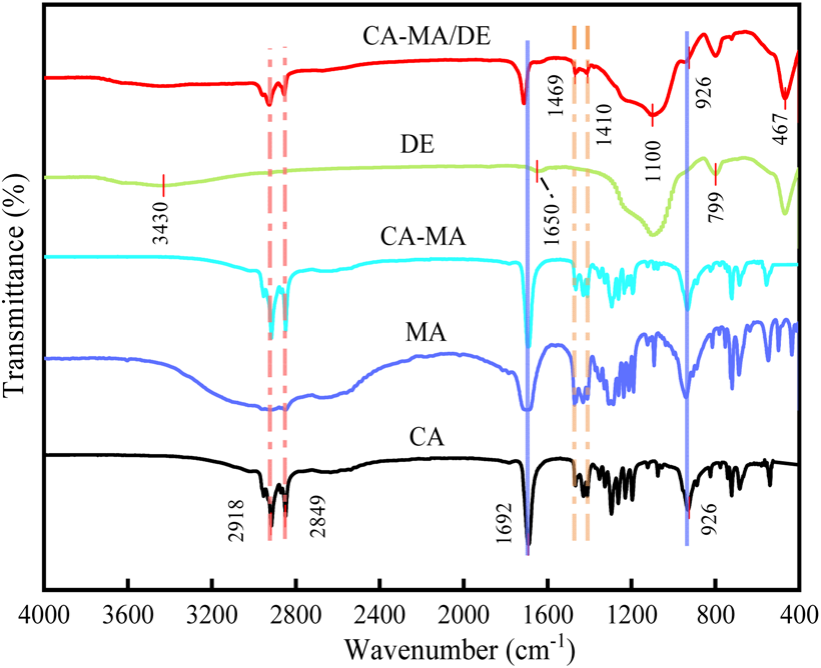
FTIR spectra of CA, MA, CA–MA, DE, and CA–MA/DE.

Fig. 3 shows the FTIR spectrum of pure CA, MA, CA–MA, DE, and CA–MA/DE. The infrared spectrum of CA appeared symmetrical stretching vibration peaks of –CH_3_ and –CH_2_ groups at 2918 and 2849 cm^−1^, the peak at 1692 cm^−1^ belongs to the C=O stretching vibration, and the peaks at 1469 and 1410 cm^−1^ were attributed to the in-plane bending vibration of –OH functional group,^19^ and the out-of-plane bending vibration peak of –OH functional group was observed at 926 cm^−1^. The diffraction peaks of MA and CA are similar, which may be because the components are the same. The peaks of CA and MA can be found in the spectra of CA–MA, and no new peaks are generated, demonstrating that there was no chemical reaction between them. As for DE, the peak was interpreted as the bending and stretching vibration of the –OH functional group near 3430 and 1650 cm^−1^, and the absorption peak at 1100 cm^−1^ was generated by the stretching vibration of the Si–O–Si group. The peak at 799 cm^−1^ represents the symmetric vibration of the Si–O–H group, and the peak near 467 cm^−1^ corresponds to the bending vibration of the Si–O group. Significantly, the FTIR spectrum of the CA–MA/DE contains both the characteristic peaks of DE and CA–MA. The shifts or broadening change in the peak intensity of CA–MA/DE may be related to weak hydrogen bonds or physical interactions when CA–MA and DE are bound. There were no obvious new peaks in the CA–MA/DE, indicating strong chemical compatibility between CA–MA and DE during the vacuum impregnation process.

### 3.2 Thermal performance

**Fig. 4:**
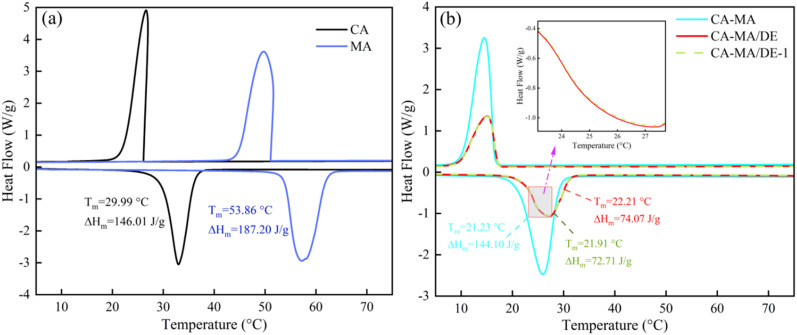
The DSC curves of (a) pure CA and MA, (b) CA–MA, CA–MA/DE, and CA–MA/DE-1.

The thermal characteristics of CA, MA, CA–MA, CA–MA/DE, and CA–MA/DE-1 were studied through the DSC analysis, with the findings presented in Fig. 4. In Fig. 4a, the DSC curves of pure CA and MA show a typical phase change process, with one endothermic and exothermic peak. The phase change temperatures of CA and MA were 29.99 and 53.86 °C, and the latent heats were 146.01 and 187.20 J g^−1^, respectively. It can be seen from Fig. 4b that the phase change temperature and latent heat of CA–MA were 21.23 °C and 144.10 J g^−1^, respectively. Compared with CA and MA, it is obvious that the phase change temperature of CA–MA has been significantly reduced, which might be observed *via* the fact that the negative shift between CA and MA. The DSC curves of CA–MA/DE are similar to CA–MA, with one peak appearing during both the heating and exothermic processes, indicating that the phase change behavior of CA–MA/DE relies primarily on CA–MA. The melting temperature and the latent heat of CA–MA/DE were 22.21 °C and 74.07 J g^−1^, respectively. The melting temperature increased by 0.98 °C compared with CA–MA, which is due to the pore restriction of DE on CA–MA, which affects the distribution and heat conduction of CA–MA, increasing phase transition temperature. The latent heat value decreased by 48.6% compared with CA–MA, which was attributed to DE is inert and does not store latent heat, and the interaction between CA–MA and DE hinders the crystallization of CA–MA and reduces the latent heat of CA–MA/DE. Moreover, the thermal performance of CA–MA/DE shows minimal changes even after 100 thermal cycles, with variations of only 0.3 °C for melting temperature and 1.36 J g^−1^ for latent heat, indicating good thermal cycling reliability. As shown in Table 2, the prepared CA–MA/DE in this study is superior to other DE-based PCMs and shows better potential application in building energy conservation.

**Table 2:** Comparison of DE-based PCMs

Samples	Melting temperature (°C)	Latent heat (J g^−1^)	Loading capacity (%)	Reference
Stearic acid/DE	52.30	57.10	28.9	40
Lauric–stearic acid/DE	31.16	59.60	35.7	41
Lauric acid/DE	40.90	57.40	40.5	42
Paraffin/DE	41.11	75.50	45.6	43
CA–MA/DE	22.21	74.07	51.4	This paper

### 3.3 Thermal stability and temperature response capacity

**Fig. 5:**
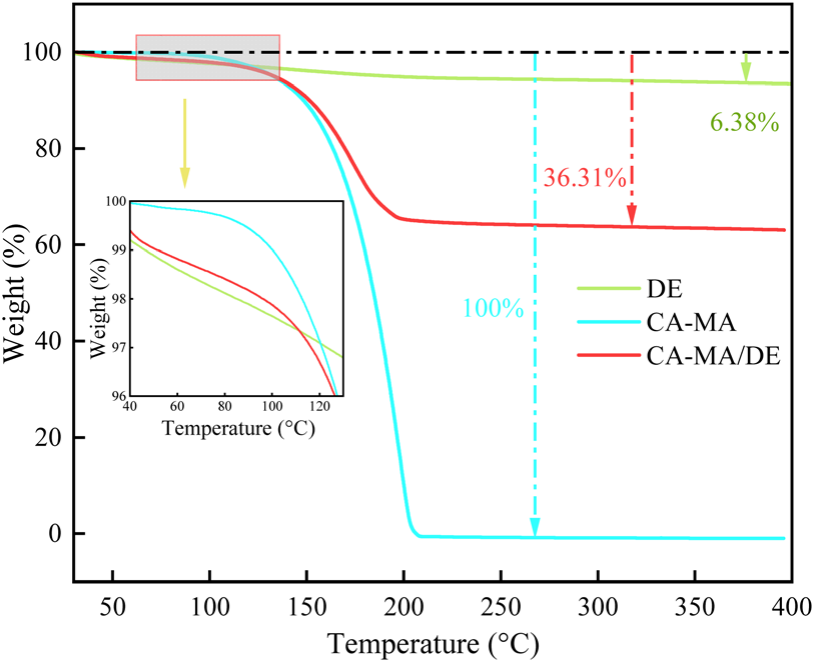
The TGA curves of DE, CA–MA, and CA–MA/DE.

TGA was used to investigate the thermal degradation and stability of CA–MA/DE. Fig. 5 shows the TGA curves of DE, CA–MA, and CA–MA/DE. The 6.38% mass loss of DE at 400 °C may be caused by water evaporation. It can be observed that CA–MA and CA–MA/DE exhibit a typical one-step thermal degradation behavior during the heating process. The mass of CA–MA was lost almost completely without any residual char, while the mass loss rate of CA–MA/DE was 36.31%. The weight loss of CA–MA began at about 92 °C and was complete at about 204 °C, which was possibly attributed to the degradation and volatilization of CA–MA. The slight loss of CA–MA/DE within 110 °C was mainly attributed to the moisture evaporation of DE, and no obvious thermal decomposition reaction was observed. The CA–MA/DE began to lose weight significantly when the temperature exceeded 110 °C, which can be ascribed to the decomposition of CA–MA. The onset thermal decomposition temperature of CA–MA was lower than that of CA–MA/DE composite, showing that DE can delay the weight loss temperature of the composite and has a better protective effect for CA–MA. It is worth noting that the little mass loss of CA–MA/DE (within 5% weight loss) at temperatures below 120 °C indicates that CA–MA/DE shows better thermal stability in the temperature range. The results show that the thermal stability of CA–MA/DE can be improved compared with CA–MA and can be used in the field of building energy conservation.

**Fig. 6:**
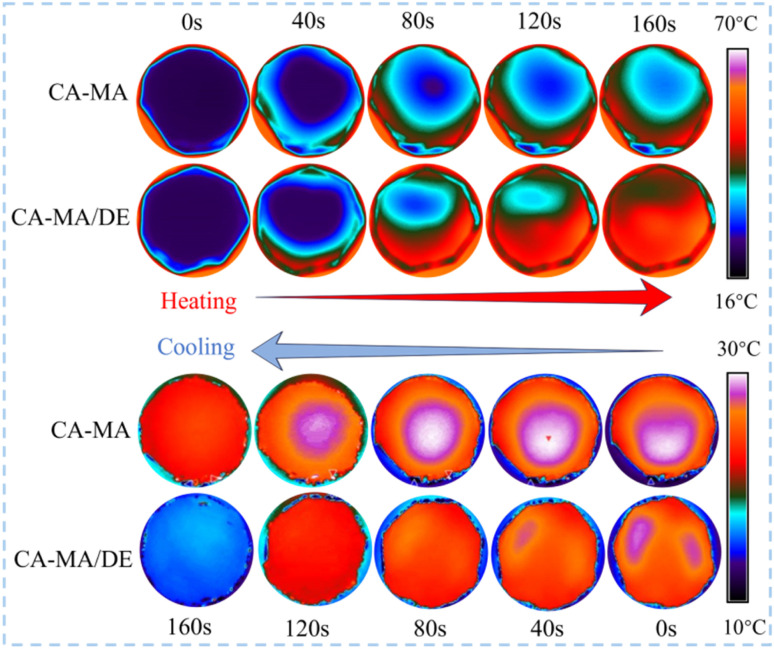
The surface temperature change image of CA–MA and CA–MA/DE.

Fig. 6 reflects the surface temperature change images of CA–MA and CA–MA/DE during the heating and cooling process. The surface temperatures of CA–MA and CA–MA/DE show different trends at different times. At the beginning of the heating process (0–40 s), there is little difference between the initial surface temperatures of CA–MA and CA–MA/DE. At 80 s, the surface temperatures of CA–MA and CA–MA/DE show obvious differences, and the temperature change of CA–MA/DE is faster than that of CA–MA. The central temperature of CA–MA/DE was heated to 42 °C at 160 s, while the temperature of CA–MA was about 31 °C. It is worth noting that the CA–MA/DE also showed better heat transfer rate during the cooling process. The temperature of CA–MA/DE was about 31 °C at 0 s and dropped to 18 °C at 160 s, while the temperature of CA–MA changed slowly between 0 and 160 s. When the center temperature of CA–MA/DE almost dropped to the ambient temperature, CA–MA could still maintain a relatively higher temperature at 160 s. The result shows that CA–MA/DE has better transient temperature response than CA–MA during the heat storage and heat release process.

### 3.4 The mechanical properties

**Fig. 7:**
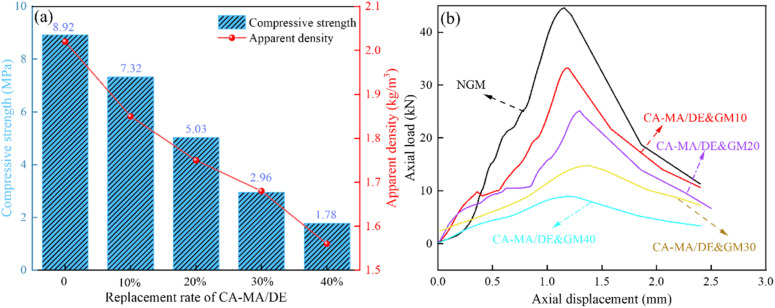
The mechanical properties (a) and load-displacement curves (b) of the samples.

Fig. 7 shows the effect of the CA–MA/DE content on the mechanical properties of NGM and CA–MA/DE&GM. Fig. 7a displays the compressive strength and apparent density curve of NGM, CA–MA/DE&GM10, CA–MA/DE&GM20, CA–MA/DE&GM30, and CA–MA/DE&GM40. The compressive strength and apparent density of NGM are the largest, which are 8.92 MPa and 2.02 kg m^−3^, respectively, it will decrease with the increase of CA–MA/DE content. This observed phenomenon may be related to several key factors. Firstly, unlike sand, the softer CA–MA/DE is less effective in improving the overall strength of gypsum mortar materials. Secondly, the addition of CA–MA/DE may disturb the initially well-graded properties of sand, thus affecting the interfacial bonding of colloids and resulting in increased porosity and decreased density in mortar. When the CA–MA/DE content reaches 30%, the compressive strength of CA–MA/DE&GM30 decreases to 2.96 MPa. When the CA–MA/DE content exceeds 30%, the compressive strength of CA–MA/DE&GM40 decreases to 1.78 MPa. The results indicated that when the CA–MA/DE content is 30% or lower, the CA–MA/DE&GM can meet the strength requirements (GB/T28627-2023, 2.5 MPa). In Fig. 7b, the load–displacement curves of NGM and the CA–MA/DE&GM with different CA–MA/DE substitution proportions were obtained from compressive experiment results. Before reaching the peak stress, the bearing capacity of all samples gradually increases with the movement of strain. When the axial load reaches the peak, the bearing capacity of CA–MA/DE&GM gradually decreases with the additional amount of CA–MA/DE, which may be because the pores were gradually compacted under the load, thus leading to the loss of the ability to resist deformation. The results show that the proper addition of CA–MA/DE can make CA–MA/DE&GM show plastic failure, which is beneficial to resist deformation and has higher reliability in engineering applications.

### 3.5 Humidity regulation ability

**Fig. 8:**
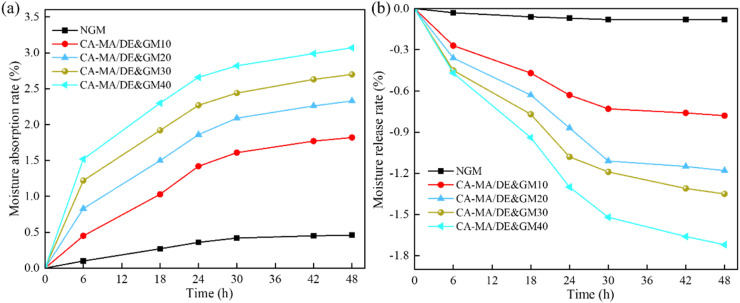
The moisture absorption (a) and release (b) properties of the samples.

Fig. 8 depicts the moisture absorption and release properties of NGM and the CA–MA/DE&GM with different CA–MA/DE incorporation amounts in the constant humidity environment during the absorption and release moisture process. Fig. 8a illustrates the change curves of the absorption moisture rate of the samples. In the high-humidity environment (RH = 100%), the moisture absorption rates of NGM and CA–MA/DE&GM increased with time within first 30 h during the moisture absorption process and gradually tended to saturation when it reached 48 h. It can be seen that NGM shows weak hygroscopicity, and the maximum moisture absorption rate was 0.46%. The moisture absorption equilibrium was observed after 30 h, and the moisture absorption rate of the whole process is less than 0.5%. The moisture absorption capacity of the CA–MA/DE&GM increased with the growth of the proportion of CA–MA/DE, which was the fastest within the first 6 h and gradually slowed down over time. At 48 h, the moisture absorption rate of CA–MA/DE&GM40 was 3.07%, showing strong moisture absorption ability. In addition, the maximum hygroscopicity of CA–MA/DE&GM30, CA–MA/DE&GM20, and CA–MA/DE&GM10 were 2.7%, 2.33%, and 1.82%, respectively. The moisture absorption rate of CA–MA/DE&GM did not reach saturation compared with NGM, and there was still a weak growth trend. It can be attributed to the fact that after the replacement of sand by the CA–MA/DE, pores will be generated at the interface connection between the DE and GM matrix, contributing to the moisture transfer, and the moisture absorption performance of the GM was enhanced. Fig. 8b represents the change curves of the moisture release rate of the samples. The CA–MA/DE&GM with different CA–MA/DE contents quickly releases moisture in a low-humidity environment (RH = 11%), and the curves gradually tend to balance during the moisture release process. The moisture release performance of NGM is poor, and it quickly reaches saturation after 30 h, with a maximum humidity release rate of 0.08%. CA–MA/DE&GM has better moisture release rate, and the content of CA–MA/DE is higher, the faster the CA–MA/DE&GM releases moisture. It can be seen that CA–MA/DE&GM30 exhibits excellent humidity control performance during moisture release, with the moisture release rate of 1.35%. It meets the application requirements while ensuring the strength requirements.

### 3.6 Heat storage and release performance

**Fig. 9:**
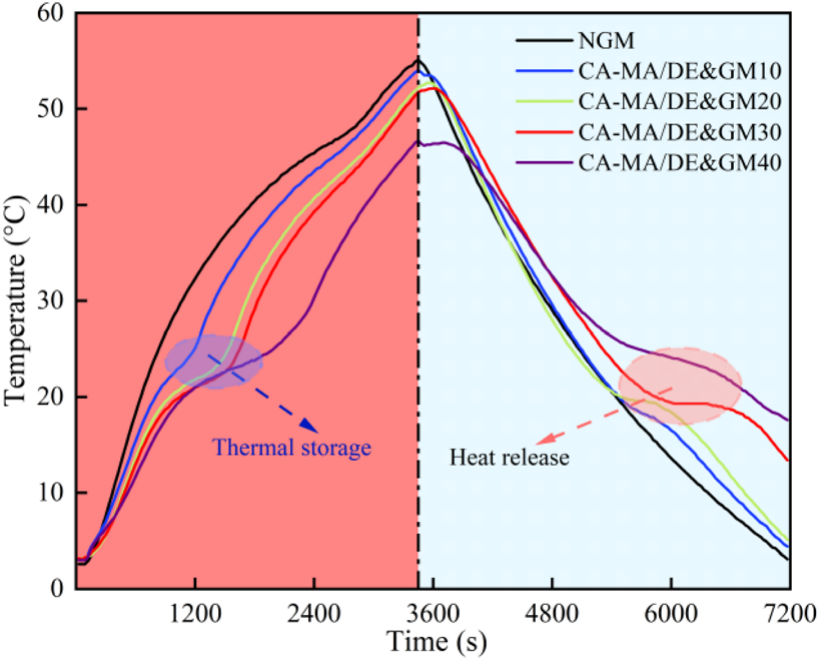
The heat storage and release properties of NGM and CA–MA/DE&GM.

The thermal energy storage and release capacity of NGM and the CA–MA/DE&GM with different CA–MA/DE incorporation amounts were tested using a constant-temperature water bath method to explore the temperature-controlled performance. Fig. 9 illustrates the temperature change curves of the samples during the heating and cooling process. It can be observed that the heat absorption rate of NGM is faster than that of CA–MA/DE&GM during the constant heating process, which may be attributed to the thermal buffer effect of the latent heat absorption of CA–MA/DE. The temperature of NGM could be reached 55.1 °C at 3400 s. Meanwhile, the temperatures of CA–MA/DE&GM10, CA–MA/DE&GM20, CA–MA/DE&GM30, and CA–MA/DE&GM40 were lower than that of NGM, which can reach 54.0 °C, 52.2 °C, 51.7 °C, and 46.7 °C, respectively. It is worth noting that the CA–MA/DE&GM with different CA–MA/DE incorporation amounts appeared in one phase change plateau during the heating and cooling process, and the temperature fluctuation was effectively slowed down. The results show that the CA–MA/DE&GM presented better temperature control performance, and the heat transfer capacity gradually increased with the incorporation of CA–MA/DE content. When the replacement rate of CA–MA/DE reaches 30%, CA–MA/DE&GM30 can delay the temperature fluctuation during the heating and cooling process, exhibiting good thermal regulation performance. It can be attributed to the thermal buffer effect of the phase change platform after adding CA–MA/DE. Meanwhile, after mixing with gypsum mortar, CA–MA/DE changes the heat transfer path of phase change materials and reduces the thermal conductivity of the mortar. Considering the mechanical, humidity control, and thermal properties of the CA–MA/DE&GM comprehensively, the ideal replacement ratio of CA–MA/DE was determined to be 30%.

### 3.7 Microscopic morphology

**Fig. 10:**
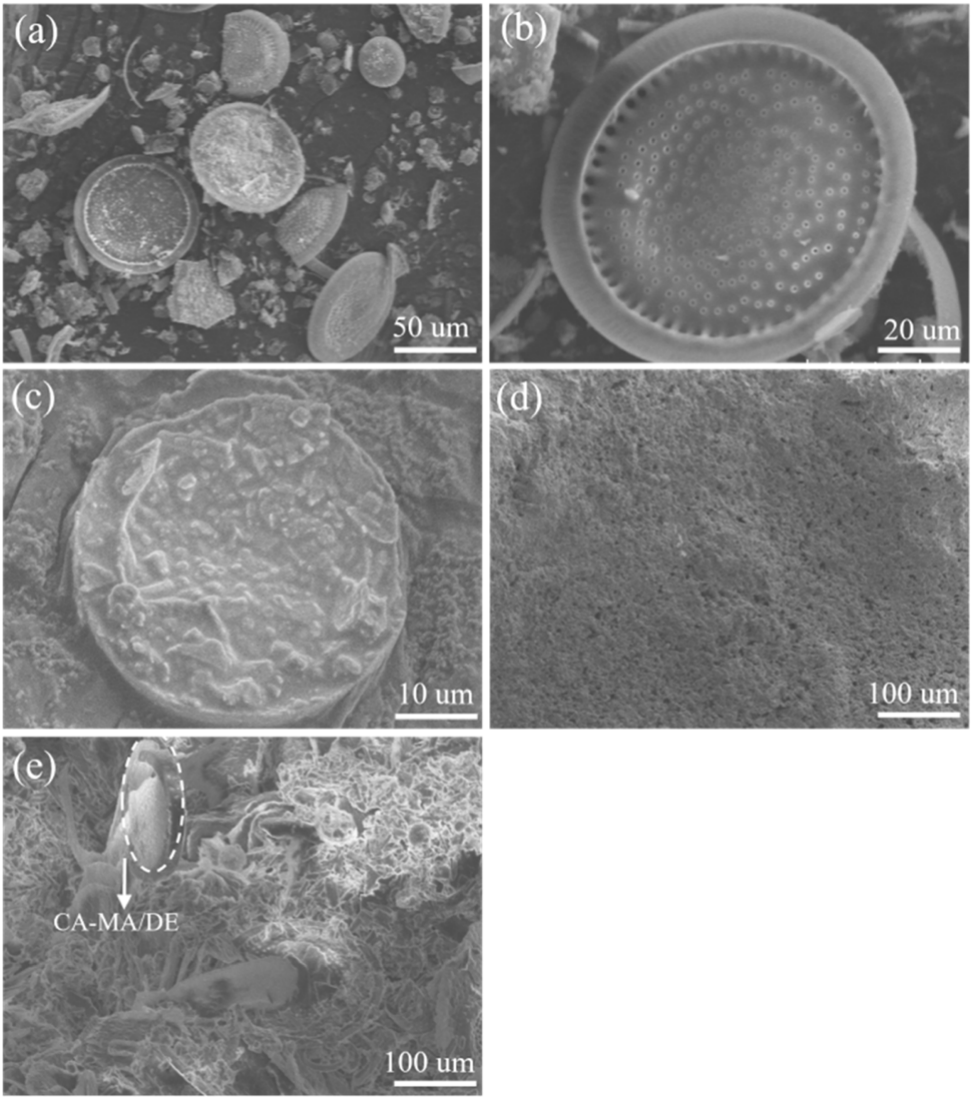
The SEM images of (a), (b) DE, (c) CA–MA/DE, (d) NGM, and (e) CA–MA/DE&GM30.

The surface morphology of DE, CA–MA/DE, NGM, and CA–MA/DE&GM30 was shown in Fig. 10. From Fig. 10a and b, it can be seen that DE presents a disk-like porous structure with abundant pores and larger specific surface area, which is conducive to the adsorption of CA–MA and the regulation of moisture. In Fig. 10c, it can be seen that CA–MA was successfully impregnated into the pores of DE. Significantly, CA–MA can be adsorbed and fixed in DE due to the surface tension and capillary force, which effectively prevents liquid phase leakage during the phase change process, thereby significantly enhancing the shape stability of CA–MA/DE. Furthermore, the morphology of CA–MA/DE still maintains a disc-like structure, further indicating that CA–MA and DE have good compatibility. From Fig. 10d, we can perceive that the surface of NGM is relatively dense and has fewer pores, which is not conducive to the permeability of water. Fig. 10e shows the image of the CA–MA/DE&GM30. It is observed that the porosity of the sample increases due to the addition of CA–MA/DE, which directly leads to the decrease of compactness. It can also explain the declining trend of compressive strength of CA–MA/DE&GM compared with NGM. However, the image of CA–MA/DE can be observed in CA–MA/DE&GM30, and there is no obvious interface gap between CA–MA/DE and the mortar matrix, which shows that it has good compatibility with cementitious materials.

### 3.8 Strain evolution of NGM and CA–MA/DE&GM30

**Fig. 11:**
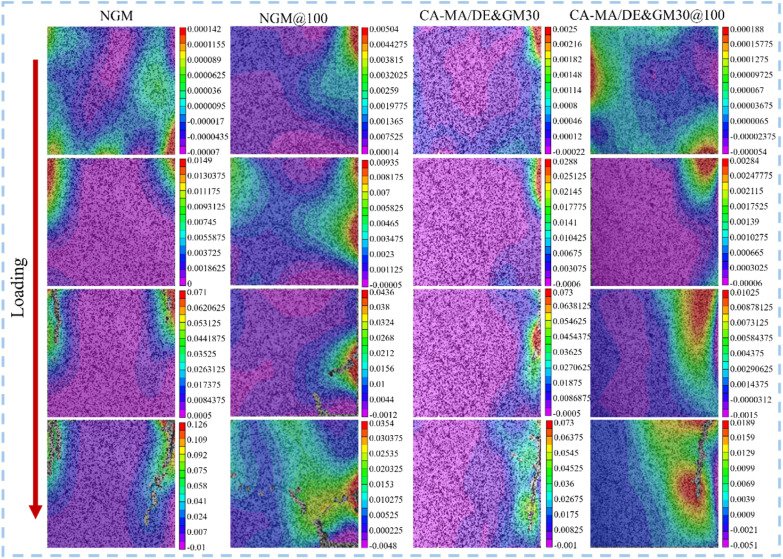
The strain cloud diagram of NGM and CA–MA/DE&GM30.

Fig. 11 shows the strain evolution mechanism of CA–MA/DE&GM30, where the red area denotes the tensile strain and the purple area signifies the compressive strain. NGM and CA–MA/DE&GM30 after 100 phase change cycles were named NGM@100 and CA–MA/DE&GM30@100, respectively. In the initial loading stage, the strain distribution of NGM, NGM@100, CA–MA/DE&GM30, and CA–MA/DE&GM30@100 is irregular, with no visible cracks in the cloud images, which could be due to the samples having a certain load-bearing capacity before damage occurs. Additionally, the internal pores of the specimens can effectively transfer stress when compressed, weakening the adverse effects caused by strain concentration. NGM shows obvious stress concentration and rapid cracking, which may be because of the compact structure and large capillary negative pressure between pores, which is not conducive to the migration of inside water, leading to the rapid growth of deformation capacity, and is typical of brittle failure. Compared with NGM, CA–MA/DE&GM30 has good deformation resistance and maintains better surface integrity under load. It may be because the pore structure of GM is loosened due to the addition of CA–MA/DE, facilitating water migration. Additionally, the hardening of GM was delayed by the moisture-regulating ability of CA–MA/DE&GM30, enhancing the compressive deformation resistance of the mortar, and ultimately delaying cracking of the sample. NGM@100 and CA–MA/DE&GM30@100 show strain concentration earlier compared with NGM and CA–MA/DE&GM30, and the number of surface micro-cracks increases obviously. Compared with NGM, CA–MA/DE&GM30 has slower micro-crack propagation and through-crack formation, showing good damage resistance. This may be because the internal temperature fluctuation of mortar can be suppressed when the ambient temperature changes after adding CA–MA/DE, and the internal defects caused by thermal expansion and cold contraction can be effectively alleviated. CA–MA/DE&GM30 has the potential to alleviate stress concentration, which provides a reference for studying the damage mechanism of mortar.

## 4 Conclusion

This study successfully developed the CA–MA/DE, which can be employed in energy-efficient construction. The chemical compatibility of CA–MA/DE was certified through the FTIR technique. The leakage experiment results show that the maximum load rate of CA–MA was determined to be 40%. The SEM analysis demonstrates that both CA–MA/DE and cementitious materials exhibit good compatibility, which maintains the internal structural integrity of the CA–MA/DE-based GM. Considering the mechanical performance, humidity regulation capacity, and thermal properties of CA–MA/DE-based GM, it has been determined that the optimum replacement rate of CA–MA/DE is 30%. The DIC results verify that CA–MA/DE&GM30 exhibits slower micro-crack propagation and through-crack formation, demonstrating good damage resistance compared to NGM, which could provide greater feasibility for energy-saving buildings.

## Conflicts of interest

There are no conflicts to declare.

## Data Availability

The data supporting this article have been included as part of the supplementary information (SI).
